# Rehabilitation to Complement Unique Single-Stage Core Decompression and Total Hip Arthroplasty in Bilateral Avascular Necrosis: A Case Report

**DOI:** 10.7759/cureus.52914

**Published:** 2024-01-25

**Authors:** Arjavi A Pakhan, Pratik Phansopkar, Manali A Boob

**Affiliations:** 1 Musculoskeletal Physiotherapy, Ravi Nair Physiotherapy College, Datta Meghe Institute of Higher Education & Research, Wardha, IND

**Keywords:** biomechanics, physical therapy, total hip arthroplasty, core decompression, avascular necrosis of the hip

## Abstract

Avascular necrosis (AVN), a debilitating condition characterized by bone tissue death due to inadequate blood supply, can severely impact the hip joint, leading to pain, limited mobility, and joint dysfunction. The complex blood supply and mechanical stress on the hip make it particularly vulnerable to AVN. Early detection is challenging as AVN may remain asymptomatic initially, but as it progresses, it results in severe joint degeneration. This case report outlines the management of a 38-year-old male patient with a dermatomyositis history who presented with bilateral hip pain attributed to AVN. Radiological investigations diagnosed grade 2 AVN in the left hip and grade 3 AVN in the right hip. The patient underwent core decompression for the left hip to halt disease progression and total hip arthroplasty (THA) for the right hip to alleviate pain and restore function. A structured three-week rehabilitation program was tailored to each surgical procedure, with pre-and post-treatment assessments revealing notable improvements in pain relief, range of motion (ROM), and muscle strength. This case underscores the importance of early diagnosis, personalized surgical interventions, and comprehensive rehabilitation in managing AVN in dermatomyositis patients. Physiotherapy is vital pre- and post-operatively to enhance physical function, strength, and mobility. Rehabilitation also plays a crucial role in postoperative recovery, early mobilization, and functional restoration. The multifaceted approach employed in this case highlights the need for a comprehensive strategy when managing AVN in dermatomyositis patients, providing valuable insights for similar cases.

## Introduction

Osteonecrosis, also known as avascular necrosis (AVN), is a debilitating disease characterized by the death of bone tissue due to an inadequate blood supply. The hip joint is one of the most often impacted regions, and AVN can cause severe discomfort, decreased range of motion (ROM), and eventually, joint dysfunction [1]. Due to its intricate vascular supply and significant mechanical stress, the hip is especially prone to AVN. While AVN might be difficult to diagnose in its early stages because of its asymptomatic nature, when the problem worsens, significant joint degeneration may occur [2]. The two main surgical procedures that have become more popular in treating AVN of the hip are total hip arthroplasty (THA) and core decompression [3]. Core decompression is a minimally invasive procedure that involves drilling a hole into the affected area of the hip bone to relieve pressure and stimulate new blood vessel growth [4]. The goal of core decompression is to halt the progression of AVN, reduce pain, and avoid the need for more extensive surgery. This procedure is particularly beneficial when AVN is diagnosed in its early stages [5].

On the other hand, THA, commonly referred to as hip replacement surgery, is a more extensive procedure used when AVN has caused significant joint damage. The procedure entails the extraction of the affected hip joint, followed by the substitution with a synthetic joint [6]. THA is an effective solution for end-stage AVN, as it can alleviate pain and restore function, enabling patients to regain their quality of life (QOL) [7]. While these surgical interventions play a vital role in managing AVN of the hip, physiotherapy is crucial in both the preoperative and postoperative phases [8]. Physiotherapy is a multidimensional approach that aims to improve the hip joint's physical function, strength, and mobility [9,10]. The preoperative phase can help patients prepare for surgery by strengthening the surrounding musculature and improving the range of motion [11,12]. In the postoperative phase, physiotherapy continues to be invaluable. For patients who have undergone core decompression or THA, it is essential to promote the healing process and regain optimal function [13]. Physiotherapists design rehabilitation programs tailored to the specific surgical procedure. They guide patients through exercises to rebuild strength in the affected hip and improve their overall mobility [14]. Additionally, physiotherapy assists in gait training, helping individuals regain a natural walking pattern [15]. By doing so, patients can adapt to their new hip joint or the changes brought about by core decompression, ultimately enhancing their long-term outcomes and QOL [16,17].

## Case presentation

Patient information

A 38-year-old male with a known history of dermatomyositis was treated with IV and oral steroids one and a half years back for six months with methylprednisolone. He presented complaints of pain in the right hip region. The pain was insidious in onset, progressive in nature, and had started one and a half months ago. Over the last five days, the pain became more intense and began radiating to his right thigh. The patient reported difficulty in climbing stairs and was unable to sit cross-legged due to the pain. The patient underwent investigations, which revealed AVN affecting both hips. In the left hip, AVN was diagnosed according to the Ficat and Arlet classification, grade 2 (sclerotic and cystic lesion), and it was managed through core decompression. In the right hip, grade 3 (flattening of the femoral head) AVN was diagnosed, and the patient underwent THA to address this condition.

Clinical findings

**Figure 1:**
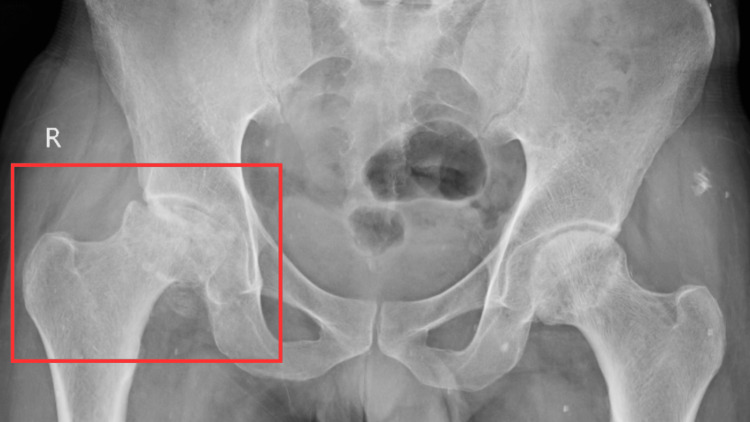
X-ray of the pelvis The red rectangle shows the destruction of the right femoral head

The patient was examined supine lying with shoulders at the same level. Both hips were slightly abducted, knees were extended, and ankles were mid-plantarflexion. On palpation, tenderness was grade 2, present over the right hip joint; there was also restricted ROM, shown in Table 1. Muscle muscle testing (MMT) revealed weakness in the hip abductors and flexors. Patient pre- and post-treatment MMT is mentioned in Table 2. X-ray of the pelvis with both hips revealed reduced joint space in the right hip joint, as shown in Figure 1. There was loss of femoral contour of the right-side femoral head and arthritic changes in the right hip joint. Magnetic resonance imaging (MRI) of bilateral hip with pelvis revealed that there was evidence of a crescent-shaped geographical area in the anterosuperior aspect of the right and left femoral head and neck appearing iso-intense on T1 weighted image/T2 weighted image/short-tau inversion recovery (T1WI/T2WI/STIR) sequences with double line sign. T2/STIR hyperintensity was noted in the femoral head as a sign of marrow edema. AVN of bilateral hip joint grade 3 on the right and grade 2 on the left (Ficat and Arlet classification) were noted. Marrow edema was noted in the bilateral femoral head, moderate joint effusion in the right hip, and mild joint effusion in the left hip. The timeline of the events is mentioned in Table 3.

**Table 1:** Range of motion (ROM)

ROM	Pre- treatment		Post- treatment	
Hip	Right	Left	Right	Left
Flexion	0-10º	0-20º	0-50º	0-60º
Extension	0-5º	0-10º	0-20º	0-25º
Abduction	0-10º	0-10º	0-25º	0-30º
Knee				
Flexion	0-30º	0-35º	0-45º	0-50º

**Table 2:** Manual muscle testing (MMT)

MMT	Pre-treatment			Post-treatment		
Muscles	Right		Left	Right		Left
Hip						
Flexors	2/5		2/5	4/5		4/5
Extensors	2/5		2/5	4/5		4/5
Abductors	2/5		2/5	4/5		4/5
Knee						
Extensor	2/5		2/5	4/5		4/5
Flexors	2/5		2/5	4/5		4/5
Ankle						
Dorsiflexors	4/5	4/5		5/5	5/5	
Plantarflexors	4/5	4/5		5/5	5/5	

**Table 3:** Timeline

Events	Date
The patient admitted to the hospital	11/10/23
Diagnosed with AVN of both hips (grade 2 in the left hip and grade 3 in the right hip)	12/10/23
Pre-operative physiotherapy commencement	13/10/23
Core decompression of the left hip (operation 1)	17/10/23
Total hip arthroplasty of the right hip (operation 2)	17/10/23
Postoperative physiotherapy begins	18/10/23

Therapeutic intervention

**Figure 2:**
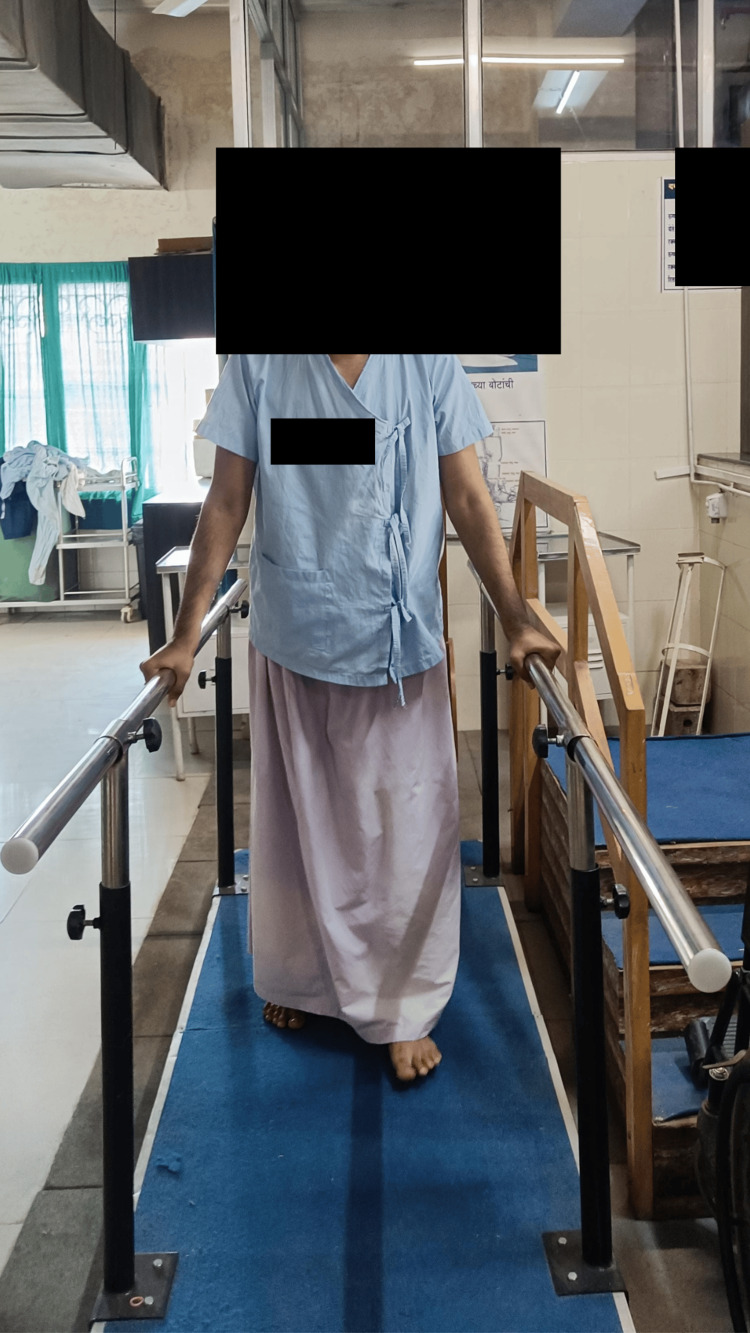
The patient walking in parallel bars

The three-week rehabilitation protocol after core decompression of the left hip is mentioned in Table 4. The three-week protocol after THA of the right hip is mentioned in Table 5. Figure 2 shows the patient performing gait training in parallel bars. 

**Table 4:** Three-week treatment protocol after the core decompression of the left hip TA: transverse abdominis; TFL:  tensor fasciae latae; ER: external rotation; IR: internal rotation

Week 1	Repetitions and duration	Rationale
Ankle and toe movements	20 repetitions, three times a day for a total of 3 weeks.	To prevent pedal edema.
Instructed in ambulation and stairs with crutches and full weight bearing	At the beginning of the physiotherapy session	Initiate gait training and promote safe mobility.
Upright stationary bike (no resistance)	20 minutes (daily)	Improves circulation, maintains joint mobility, and reduces swelling without stressing the joint.
Continuous passive movement usage	2 times per day	Continuous passive motion helps prevent joint stiffness and promotes healing.
Passive range of motion (circumduction, abduction, log rolls)	5-10 repetitions for each motion	Promotes joint mobility without stressing the hip.
Prone lying	2-3 hours/day	Reduces pressure on the hip, minimizes discomfort, and prevents complications.
Isometrics (quad sets, glut sets)	Hold each for 5 seconds, 20 repetitions for each muscle group	Maintain muscle tone and promote neuromuscular control.
Quadriped exercises (rocking, pelvic tilts, arm lifts)	2 times per day	Promote core stability and proximal control.
Clams/reverse clams	2 sets of 10 repetitions for each exercise	Activate hip muscles without overloading the joint.
TA activation with bent knee fallouts	2 sets of 10 repetitions	Enhance core and hip muscle engagement.
Bridging initiation	2 sets of 10 repetitions	Improve hip and core strength.
Week 2	Repetitions and duration	Rationale
Stationary bike (20 minutes): increase time at week 3 as the patient tolerates	20 minutes	Continue to improve circulation and joint mobility.
Soft tissue mobilization (specific focus on adductors, TFL, Iliopsoas, and Inguinal ligament)	20-30 minutes each session	Address specific soft tissue restrictions to enhance hip function.
Isometrics (quad, glutes, TA)	Hold each for 5 seconds, 20 repetitions for each muscle group	Maintain muscle strength and control.
Quadriped exercises (rocking, pelvic tilts, arm lifts)	Daily	Promote core stability and control.
Anterior capsule stretches: surgical leg off the table	10-15 seconds hold for each stretch	Continue to improve hip flexibility.
Clams/reverse clams	2 sets of 10 repetitions for each exercise	Further strengthen hip muscles.
TA activation with bent knee fallouts	2 sets of 10 repetitions	Enhance core and hip muscle engagement.
Bridging progression	2 sets of 10 repetitions	Improve hip and core strength.
Prone hip external rotation/Internal rotation, hamstring curls	2 sets of 10 repetitions	Strengthen hip muscles without compromising the joint.
Week 3	Repetitions and duration	Rationale
Stationary bike (increase time as tolerated)	As tolerated	Continue to improve circulation and joint mobility.
Soft tissue mobilization (specific focus on adductors, TFL, iliopsoas, and inguinal ligament)	20-30 minutes for each session	Address soft tissue restrictions for improved hip function.
Isometrics (quadriceps, gluteus, transversus abdominis)	Hold each for 5 seconds, 20 repetitions for each muscle group	Maintain muscle strength and control.
Anterior capsule stretches which comprise gently stretching the front part of the hip joint, promoting flexibility and mobility in the surgical leg	10-15 seconds hold for each stretch	Maintain and improve hip flexibility.
Clams/reverse clams	2 sets of 10 repetitions for each exercise	Continue strengthening hip muscles.
TA activation with bent knee fallouts	2 sets of 10 repetitions	Enhance core and hip muscle engagement.
Bridging progression	2 sets of 10 repetitions	Further improve hip and core strength.
Prone hip ER/IR, hamstring curls	2 sets of 10 repetitions	Strengthen hip muscles without compromising the joint.

**Table 5:** Three-week treatment protocol after total hip arthroplasty of the right hip TENS: transcutaneous electrical nerve stimulation

Week 1: Initial post-operative phase (days 1-7)			
Goals	Procedures	Repetitions	Rationale
Patient education	Educate the patient on hip precautions: instruct on proper body mechanics and transfers	Ongoing throughout the week	Ensures patient’s safety and adherence to precautions.
Pain management	Gentle passive range of motion exercises (flexion, extension, abduction, adduction), cryotherapy	2-3 times per day	Early pain control promotes mobility and exercise compliance.
Edema control	Ankle pumps and ice application	10-15 repetitions, 3-4 times a day, 20 minutes every 2 hours	Reducing swelling minimizes discomfort and aids in wound healing.
Complication prevention	Ankle pumps, early ambulation with assistance. Sequential compression devices	10-15 repetitions, 4-5 times a day, as tolerated and prescribed	Mobilization and ambulation help prevent complications, such as deep vein thrombosis.
Improve mobility	Gentle passive range of motion exercises. Bed exercises to avoid hip flexion beyond 90 degrees. Isometric gluteal and quadriceps contractions	2-3 times per day (as tolerated) 10-second holds, 10-15 repetitions	Gentle range of motion promotes joint healing and prevents adhesions.
Week 2: Subacute phase (days 8-14)			
Goals	Procedures	Repetitions	Rationale
Improved pain control	Passive range of motion exercises. Modalities for pain management (e.g., heat, TENS)	2-3 times per day. As prescribed	Continued pain relief enhances patient mobility.
Progressive mobility	Active assisted and active range of motion exercises. Gentle stretching exercises. Gentle resistance band exercises	2-3 times per day, 10-15 repetitions. 2-3 sets of 10-15 repetitions	Gradual increase in range of motion and strength.
Muscle strengthening	Isotonic strengthening exercises (quadriceps, hip abductors, hip extensors). Closed kinetic chain exercises	3 sets of 10-15 repetitions, 2-3 sets of 10-15 repetitions	Rebuilds muscle strength around the hip joint.
Gait training	Use of assistive devices (crutches, walker). Weight-bearing exercises. Gait training with the physical therapist	As tolerated: guided by the therapist	Focus on walking with proper weight-bearing.
Functional activities	Transfer training. Activities of daily living (e.g., sitting, standing, climbing stairs)	As guided by the therapist	Encourages patient independence in daily tasks.
Week 3: Advanced rehabilitation (days 15-21)			
Goals	Procedures	Repetitions	Rationale
Increased strength	Progressive resistance exercises (leg press, step-ups). Dynamic stability exercises: functional exercises (lunges, squats)	3 sets of 10-15 repetitions. 2-3 sets of 10-15 repetitions. As guided by the therapist	Increases strength and stability for improved function.
Improved mobility	Advanced gait training. Walking without assistive devices, with emphasis on proper gait mechanics (heel-to-toe). Progressive return to normal activities	10-15 repetitions. As tolerated	Enables regaining near-normal gait patterns for independence and confidence.
Balance training	Static balance standing on the surgical leg with support if needed. Progress to eyes-closed balancing. Weight Shifting by standing on both legs. Shift weight to the surgical leg while maintaining balance. Gradually increase the duration of weight transfer. Tandem stance standing with one foot in front of the other (heel-to-toe). Progress to eyes-closed tandem stance. Mini-squats with standing with feet shoulder-width apart. Slowly lower the body into a mini-squat position. Hold onto a stable surface if needed. Step-ups. Use a stable step or platform. Step up with the surgical leg and step down. Hold onto a rail or support for safety	Hold for 30 seconds to 1 minute, 2-3 sets. 10-15 seconds each side, 2-3 sets. Hold for 30 seconds to 1 minute, 2-3 sets. 10-15 repetitions, 2-3 sets. 10-15 step-ups, 2-3 sets	Improves single-leg stance, enhancing weight-bearing on the surgical hip. Enhances weight-bearing on the surgical hip, improving weight distribution. Challenges balance and stability in a controlled manner. Strengthens the hip and leg muscles while improving balance. Enhances strength and control in the surgical hip during step activities.

Outcome measures

The physiotherapy intervention was carried out for three weeks following the progression outcome measures, which showed improvement after the physiotherapy intervention. Outcome measures are mentioned in Table 6.

**Table 6:** outcome measures HHS: Harris hip score; LEFS: lower extremity functional scale; NPRS: numerical pain rating scale

Outcome measure	Pre-operative	Post-operative
HHS	52/100	78/100
LEFS	26/80	58/80
NPRS	9/10	3/10

## Discussion

The presented case report emphasizes the importance of early diagnosis in AVN, particularly in patients with underlying conditions like dermatomyositis. This debilitating condition, characterized by bone tissue death due to insufficient blood supply, often remains asymptomatic in its early stages, making timely detection challenging. In this case, a 38-year-old male patient with a dermatomyositis history developed bilateral AVN in his hip joints, ultimately leading to severe hip pain and limited mobility. Radiological investigations revealed grade 2 AVN in the left hip and grade 3 AVN in the right hip, highlighting the advanced stage of the disease in the latter. The choice of surgical interventions, core decompression for the left hip and THA for the right hip, was determined by the severity of AVN in each hip. Core decompression aimed to halt disease progression, while THA was used to treat the severely affected right hip to relieve pain and regain function. Physiotherapy was pivotal in managing this case, both in the preoperative and postoperative phases. Preoperative physiotherapy prepared the patient for surgery by enhancing muscle strength, improving the ROM, and optimizing joint function [18]. Postoperative physiotherapy was essential in promoting healing, rebuilding strength, and improving mobility. The tailored rehabilitation programs were designed to meet the specific needs of each surgical procedure and the patient's condition [19].

The outcomes of this case were encouraging, with the patient experiencing a significant reduction in pain, as indicated by a decrease in the Numerical Pain Rating Scale (NPRS) from 9/10 to 3/10. The ROM in both hips improved substantially, indicating better joint function, and manual muscle testing (MMT) results revealed remarkable progress in muscle strength, particularly in the hip and knee muscles. Karim and Goel thoroughly explained end-stage femoral head osteonecrosis therapy, significant causes of AVN, diagnostic concerns, and the option for cementless THA. It highlights the crucial role of radiological features in diagnosis, emphasizing the patient's extensive osteonecrosis of the right hip and the potential use of advanced imaging techniques. The text underscores the advantages of cementless THA over cemented options, particularly in younger patients, while acknowledging the need for further long-term studies. Rehabilitation's significance in postoperative recovery, early mobilization, and functional restoration is emphasized. Lastly, it stresses the importance of ongoing follow-up for patients, especially younger adults, to monitor the surgical procedure's integrity and long-term outcomes [20].

## Conclusions

This case study highlights the importance of promptly identifying and tailoring therapeutic approaches for patients with AVN who also have underlying conditions such as dermatomyositis. The patient's experience with bilateral hip AVN illustrates the importance of identifying disease progression and selecting appropriate surgical interventions to manage pain and restore function effectively. A comprehensive rehabilitation protocol, designed to reduce pain, improve joint function, restore a normal gait pattern, and enhance balance, is crucial for the patient's overall mobility and QOL.
